# The Benefits of Covert Retrieval Practice on Short- and Long-Term L3 Vocabulary Retention

**DOI:** 10.3390/bs16071124

**Published:** 2026-07-06

**Authors:** Siwei Long, Yijie Wang, Jiaxuan Chen, Hongwen Cao, Yi Tang

**Affiliations:** 1 Research Center for Language, Cognition and Language Application, Chongqing University, Chongqing 401331, China; 20240401006@stu.cqu.edu.cn (S.L.); m18716629311@163.com (Y.W.); 202504106771@stu.cqu.edu.cn (J.C.); 2 Department of English, Faculty of Arts and Humanities, University of Macau, Macau 999078, China

**Keywords:** retrieval practice, third language acquisition, Japanese, metacognitive judgments

## Abstract

Background: The present study investigated the impact of retrieval practice on Japanese vocabulary acquisition by native Chinese speakers (English as the second language), employing a classic retrieval practice paradigm and examining both short-term and long-term effects. Methods: Fifty-eight Chinese participants without prior knowledge of Japanese learned 30 Japanese–Chinese word pairs through either repeated studying or retrieval practice. Participants subsequently provided metacognitive predictions before completing a 15-min delayed cued recall test, as well as 30-day and 50-day delayed old/new recognition memory tests. Results: Retrieval practice with feedback significantly enhanced vocabulary recall on the 15-min cued recall test and recognition on the 30-day and 50-day tests compared to repeated studying. Regarding metacognitive judgments, participants’ predicted performance significantly declined over longer retention intervals, but no significant differences were found between retrieval practice and repeated studying at any retention interval. Conclusions: The findings provide behavioral evidence for the advantage of retrieval practice in kana-based (phonographic) L3 vocabulary learning, extending the applicability of the desirable difficulties framework and the retrieval effort hypothesis to this context. The study also highlights the need to raise the awareness of retrieval practice among learners and educators. Future research should explore the generalizability of these findings to logographic (kanji) learning and to more ecologically valid educational settings.

## 1. Introduction

A substantial body of research has demonstrated that retrieval practice is an effective method for enhancing memory performance (Candry et al., 2020; Karpicke & Roediger, 2008; Li et al., 2024). In the domain of second language (L2) acquisition, retrieving information from memory has been shown to improve test performance and facilitate long-term retention, a phenomenon commonly referred to as the retrieval practice effect or testing effect (Karpicke & Zaromb, 2010; van den Broek et al., 2016; Rowland, 2014). The desirable difficulty framework (Bjork, 1994) posits that a moderate level of difficulty can stimulate learners to engage in deeper cognitive processing, thereby enhancing long-term memory retention. Building on this, the retrieval effort hypothesis proposed by Pyc and Rawson (2009) suggests that the effort expended during retrieval is a key factor in improving memory, emphasizing that greater effort during successful retrieval yields stronger memory benefits. Furthermore, the elaborative retrieval hypothesis (S. K. Carpenter, 2009, 2011) describes a more specific cognitive mechanism, arguing that retrieval attempts broaden the semantic network linked to the target information, and this semantic elaboration is fundamental for durable memory. Empirical studies in L2 acquisition have provided broad support for these theoretical frameworks (Karpicke & Roediger, 2008).

Research in this field typically employs a classic three-phase experimental paradigm to isolate and quantify the retrieval practice effect. Firstly, all items are studied during the initial learning phase. Subsequently, in the intervention phase, a subset of items is practiced via retrieval attempts (often with feedback), while the remaining items are subjected to repeated study. Finally, in the final test phase, the memory retention of all items is assessed. The provision of immediate feedback is a common and crucial design feature, as substantial evidence indicates that retrieval practice with feedback maximizes mnemonic benefits compared to retrieval without feedback (Butler & Woodward, 2018; Rowland, 2014). The strength of the practice effect is measured by comparing performance differences between the retrieval practice condition and the repeated study condition on the final test (Rickard & Pan, 2018).

However, despite the robustness of this paradigm, several limitations persist as research seeks to extend its application. Firstly, its empirical foundation is predominantly built on European alphabetic languages (e.g., English), while logographic systems such as Chinese characters and Japanese kanji have received less attention. This limitation is theoretically significant, as cognitive models of reading posit fundamental processing differences between these systems. Alphabetic languages are thought to follow a “multiple-route interactive model”, where lexical processing is influenced by the interaction of orthography, phonology, and semantics (Kuperman et al., 2009). In contrast, logographic systems exhibit more direct orthography-to-semantics mapping, relying more heavily on holistic visuospatial analysis rather than phonological decoding (Biederman & Tsao, 1979). Consequently, if the retrieval practice effect operates primarily through phonological processing, different patterns might be observed in logographic systems.

Research on retrieval practice in alphabetic L2 acquisition, such as English, has yielded several well-established findings. Studies indicate that retrieval practice significantly promotes the long-term retention of English vocabulary, with retrieval formats such as free recall and cued recall generally producing stronger effects than recognition tests (S. K. Carpenter & DeLosh, 2006; Rowland, 2014). Furthermore, feedback plays a pivotal role in this process; immediate feedback can amplify the positive effects of retrieval practice by strengthening phoneme-to-semantic mappings (Butler & Woodward, 2018; Kang et al., 2007).

A limited number of studies on logographic L2 acquisition have provided preliminary evidence for retrieval practice effects. For example, Kang (2010), using English-native speakers, demonstrated that “retrieval practice combined with immediate feedback” enhanced long-term memory for Chinese (logographic) vocabulary more effectively than restudying. Japanese, incorporating both meaning-based kanji and phonetically-based kana, presents a relevant context for examining script-based modulation of these effects, although the present study focuses exclusively on kana (Otsuka & Murai, 2020). Ignoring such scriptal differences risks theoretical overgeneralization and pedagogically ineffective implementations.

Secondly, the vast majority of retrieval practice research has focused on L2 acquisition contexts (Candry et al., 2020; Kanayama & Kasahara, 2016; Karpicke & Roediger, 2008; van den Broek et al., 2013), with only scarce empirical attention paid to third language (L3) acquisition (Li et al., 2022b; Tse & Pu, 2013). Core findings from L2 research—such as the superiority of retrieval practice over restudying and the feedback enhancement effect—have been validated across different language backgrounds, and the underlying cognitive mechanisms (e.g., semantic elaboration, cognitive effort) demonstrate cross-linguistic generality (S. K. Carpenter, 2011; Karpicke & Roediger, 2008). This provides a theoretical basis for extrapolating these findings to L3 contexts. However, it is important to note that L3 acquisition involves cross-linguistic influences from both L1 and L2 (i.e., potential dual interference), which may modulate the strength and specific mechanisms of the retrieval practice effect. For instance, when the L3 differs significantly from the L2 in writing system (e.g., English vs. Japanese), conclusions from L2 research regarding “phonologically-mediated retrieval effects” may not directly apply. Therefore, a focused investigation into the unique orthographic processing characteristics of the L3 is warranted, which constitutes a central aim of the present study.

Given the more complex dynamics of cross-linguistic influence (CLI) in L3 acquisition, this research gap is theoretically critical. CLI refers to the non-selective co-activation of all known languages during language processing, leading to cross-linguistic interaction (Kroll et al., 2015; Rice & Tokowicz, 2020). Compared to L2 learners who primarily suppress L1 interference, L3 learners must manage interference from both their L1 and L2 (Sudina & Plonsky, 2021). This potentially amplifies “desirable difficulty” and cognitive effort demands through more intricate cross-linguistic interplay. Furthermore, transfer in L3 acquisition can originate from both L1 and L2, with L2 often becoming the preferred source when it shares structural similarities with the L3 (Falk et al., 2015; Rothman, 2010). Although the cognitive processes in L3 acquisition are more complex, the significant typological and orthographic differences between English (L2) and Japanese (L3) likely result in weaker transfer from English to Japanese. Consequently, the behavioral manifestation of the retrieval practice effect in this context may not fundamentally differ from that observed in general L2 acquisition.

The research on retrieval practice in L3 learning has generated consistent empirical evidence (e.g., Tse & Pu, 2012; Li et al., 2022a; Huang et al., 2025). Tse and Pu (2012) suggest that retrieval practice is beneficial for long-term memory and cross-linguistic transfer, and Li et al. (2022b) reported the enhanced effect of feedback on retrieval practice and emphasized the asymmetry between L1–L3 and L2–L3 pairings. Recent research further suggests that this influence is robust and may increase with additional learning rounds (Huang et al., 2025).

However, this apparent trial-dependent gain is not consistently observed. Under a one-week delay with recognition testing, increasing learning trials beyond three failed to yield additional benefits (Huang & Cao, 2026), indicating that the role of learning trials is conditional rather than general. This inconsistency points to unresolved boundary conditions, particularly regarding retention interval and test format.

More broadly, the literature is constrained in several ways. Retention intervals are typically short, limiting claims about long-term durability. Methodologically, the heavy reliance on measures of recall may underestimate residual memory over extended delays, whereas assessments of recognition, arguably more sensitive to consolidation, remain underused. In addition, metacognitive dimensions are largely neglected: the sparse inclusion of judgments of learning (JOLs) leaves unclear how retrieval practice shapes learners’ monitoring and control processes.

A further, underexplored issue concerns L3 acquisition in contexts where the L1 and L3 share orthographic features. For native Chinese speakers learning Japanese, the existence of cognate kanji characters can lead to “parasitic lexical association” (Kato, 2005)—that is, learners rely on orthographic similarity to infer meaning, which in turn creates semantic interference and may undermine long-term retention (Ecke, 2015; Otwinowska & Szewczyk, 2019). Hence, to obtain a clear picture of the retrieval practice effect, it is necessary to control for this variable.

To address the aforementioned limitations, the present study systematically investigates the impact of retrieval practice on vocabulary learning and long-term retention within the specific context of Japanese as an L3 for native Chinese speakers. To dissociate the effect from kanji-mediated transfer, we exclusively employ kana-scripted vocabulary as experimental materials, a choice aligned with findings that memorization strategies differ for kanji and kana (Mariko et al., 2001). Because native Chinese speakers have no prior exposure to kana, this design effectively controls for positive transfer from their L1 (Chinese), creating a purer test of the retrieval practice effect unconfounded by pre-existing orthographic knowledge. Additionally, learning an unfamiliar syllabary demands substantially more cognitive effort than learning alphabetic scripts, which aligns with the desirable difficulty framework (Bjork, 1994): the moderate difficulty introduced by kana materials may stimulate deeper processing, thereby enhancing long-term retention. We adopted the standard three-phase retrieval practice paradigm (Rickard & Pan, 2018). During the intervention phase, participants in the retrieval practice condition engage in recall attempts followed by immediate feedback. In the context of paired-associate learning, productive learning (L1 → L3) refers to producing the target L3 word from an L1 cue, whereas receptive learning (L3 → L1) refers to recognizing the meaning of an L3 word from its form (Webb, 2005). The present study adopts productive learning in the retrieval practice condition to increase cognitive effort.

Unlike studies focusing primarily on working memory or short-term retention, this research emphasizes the long-term retention of lexical knowledge. Long-term memory, characterized by its persistence and large capacity (Camina & Güell, 2017), enables learners to better retrieve and apply knowledge in real-world language use—a primary goal of language learning. A distinctive feature of this study is its focus not only on immediate learning outcomes but also, more importantly, on the long-term retention facilitated by retrieval practice. Our experimental design incorporates multiple delayed test points (15 min, 30 days, and 50 days post-intervention). This extends beyond the typical one-week interval common in prior research, allowing for a more thorough examination of long-term memory consolidation and decay dynamics. Given the significant memory decay associated with long intervals, typical recall tests (e.g., cued recall) may systematically underestimate the true retention levels due to their reliance on the retrieval of detailed information. Therefore, we employed a cued recall test at the immediate final test (15 min) to reliably assess knowledge acquisition. For the 30-day and 50-day delayed tests, we utilized an old/new recognition test. Recognition tests, which require only a judgment of prior occurrence and are less dependent on detailed information, are more sensitive to detecting residual memory strength after long delays, thus providing a more accurate assessment of memory consolidation (Craik & Lockhart, 1972; Yonelinas, 2002). Additionally, we integrated a metacognitive measure, namely judgments of learning (JOLs), to evaluate the participants’ monitoring and awareness of their own cognitive processes and knowledge state (Siedlecka et al., 2019). This contributes to a more comprehensive understanding of how retrieval practice influences learners’ subjective learning experiences.

Based on relevant theories of retrieval practice (S. K. Carpenter, 2011; Karpicke & Roediger, 2008; Bjork, 1994) and considering the likely weak transfer from English (L2) to Japanese (L3), we hypothesized that participants in the retrieval practice condition would demonstrate superior performance in the 15-min cued recall test and would maintain this advantage in the 30-day and 50-day old/new recognition tests. Should this study provide evidence for the significant advantage of retrieval practice in promoting long-term vocabulary retention within this specific phonographic L3 learning context, it would not only furnish new behavioral evidence for understanding memory mechanisms across different writing systems and complex multilingual hierarchies, but also offer scientifically-grounded guidance for foreign language pedagogy on leveraging retrieval practice to foster durable learning.

## 2. Methods

Fifty-eight native Chinese speakers (32 male; M = 22.60 years, SD = 2.35) from various universities participated in the study. All participants were right-handed, reported no history of reading impairments or neurological disorders, and had no prior experience in learning Japanese (self-reported). Participants’ academic majors were diverse and not restricted to any specific field. Before the experiment, written informed consent was obtained from all participants, who were compensated with either cash or course credit. The study protocol was approved by the relevant institutional ethics committee.

The study consisted of two phases. All 58 participants completed the first phase (15-min delayed test). In the second phase (30-day delayed old/new recognition test and 50-day delayed old/new recognition test), 27 participants were successfully contacted and completed the test, resulting in a retention rate of 46.6% of the original sample. Six initially recruited participants were excluded from the analysis: four due to poor performance (scores below 5 out of 15), and two for not following the experimental instructions.

### 2.1. Materials

The research materials comprised 30 pairs of Japanese kana words and their corresponding Chinese translation equivalents. Selection criteria were as follows: all selected Chinese and Japanese words were nouns, Japanese words were written in kana form and consisted of two characters; corresponding Chinese words also consisted of two characters, and these Chinese words were common and of high frequency. Stroke count was adjusted to ensure that there was no significant difference between retrieval practice and repeated study conditions. Usage frequency data for Japanese and Chinese words were sourced from NINJAL-LWP for BCCWJ (Maekawa et al., 2014) and the SUBTLEX-CH database (Cai & Brysbaert, 2010). The 30 Chinese–Japanese pairs were evenly split between the retrieval practice condition (15 pairs) and the repeated study condition (15 pairs), with a paired-sample *t*-test showing no significant condition differences in word frequency or stroke count. Specific frequency and stroke count data are detailed in Table 1.

### 2.2. Design and Procedure

A 2 × 3 mixed design (learning condition: retrieval practice vs. repeated study; retention interval: 15 min vs. 30 days vs. 50 days) was used for the study. The experimental program was written and run via E-Prime 3.0 software.

Participants performed the experimental task while seated in a quiet testing room with stimuli presented on a centrally-positioned computer monitor. Prior to the experiment, all participants provided informed consent and received compensation (cash or volunteer hours) upon completion.

**Figure 1:**
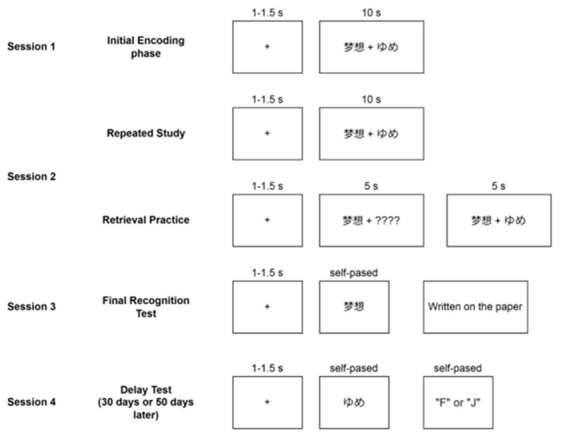
Schematic illustration of the procedure in Session 1, which involved three phases. Before each trial, a central fixation cross (+) was presented for 1–1.5 s. During the encoding phase, participants studied 30 Chinese–Japanese word pairs once (e.g., ”梦想” (Chinese, meaning “dream”) paired with its Japanese translation ”ゆめ” (Japanese kana, meaning “dream”). During the intervention phase, 15 word pairs were repeatedly studied and 15 word pairs were repeatedly retrieved. In the final test phase, a 15-min delayed cued-recall test was conducted, with Chinese cues for all 30 word pairs presented on screen. Participants recalled and wrote the corresponding Japanese words at their own pace on paper prepared in advance. The notation “????” indicates that the screen displayed “????” during that interval, and participants performed covert retrieval. Follow-up recognition tests were administered 30 and 50 days later, in which participants viewed Japanese kana on screen (e.g., “ゆめ”, meaning “dream”) and judged whether each item had been learned previously.

The first experiment session consisted of three stages: initial encoding, intervening, and final testing (Figure 1). During initial encoding, all 30 Chinese–Japanese word pairs were presented at the center of the computer screen for 10 s, with 1–1.5 s intervals between trials. After encoding, participants performed a 3-min distraction task consisting of serial subtraction: starting from 300, they repeatedly subtracted 7 and performed the subtraction mentally to reduce memory interference. Participants then proceeded to the intervening phase, where word pairs were divided between retrieval practice and repeated studying conditions. In the retrieval practice condition, participants covertly retrieved the corresponding Japanese word within 5 s (i.e., they attempted to recall it in their minds) and then received 5 s of corrective feedback. In the repeated study condition, each pair was presented for 10 s of restudying. Materials were presented in 7 randomized blocks, with each item practiced once per block, resulting in seven learning trials per item.

After a 15-min retention interval, participants wrote Japanese words on paper based on Chinese prompts, with no time limit for recall. Paper-based testing was employed because E-Prime software did not support Japanese input. It is important to note that throughout the encoding and intervention phases, kana characters were presented only as printed images; no handwriting instruction or pen-stroke modeling was provided. Participants learned the visual forms of kana through repeated exposure. In the cued recall test, they were asked to reproduce these visual forms on paper from memory, without prior practice in writing kana. Prior to the final test, participants provided judgments of learning (JOLs) for three retention intervals (15 min, 30 days, and 50 days) and for both learning conditions (retrieval practice and repeated study). For each combination of interval and condition, participants were asked to estimate how many of the 15 words they would correctly recall (on the 15-min cued recall test) or correctly recognize (on the 30-day and 50-day old/new recognition tests) by writing a number between 0 and 15 (0 = “none”, 15 = “all 15 words”). A sample prompt for the 15–min cued recall test was: “How many of the 15 words in Condition 1 (e.g., dream → ゆめ) do you think you can correctly recall and write after 15 min?”; analogous prompts were used for the other intervals and conditions. JOL data were collected from all 58 participants during the first session. After excluding participants with missing JOL ratings for any of the three time points (*n* = 19), the final JOL analysis included 39 participants.

The second session was separately scheduled at 30 days and 50 days later, when 30 unlearned Japanese words were mixed with previously learned Japanese words in an old/new recognition memory test. Participants determined whether the Japanese words were new or old by pressing “F” or “J” (counterbalanced), or the space bar if uncertain. The space bar response was scored as incorrect. Twenty-seven participants successfully completed this follow-up test.

### 2.3. Scoring and Analysis

#### 2.3.1. Scoring Protocol

To ensure objectivity in the 15-min delayed cued recall test, responses were evaluated by two Japanese Language Proficiency Test (JLPT) N1 certified raters. They judged orthographic accuracy by comparison with the printed version. Completely correct answers received 1 point, while errors or merely writing Chinese prompts received 0 points. Disagreements between raters were resolved through discussion. For the 30-day delayed old/new recognition memory test, the computer automatically scored results, with correct judgments receiving 1 point and errors or uncertain judgments receiving 0 points. All three conditions (retrieval practice, repeated study, and new words) followed the same scoring protocol.

#### 2.3.2. Analysis

All data analyses were conducted in R Studio 4.4.3 (R Core Team, 2025). We used generalized linear mixed-effects models (GLMMs) with a binomial distribution and a logit link function, implemented via the glmer function in the lme4 package (Bates et al., 2015), for all behavioral and JOL data.

For the 15-min delayed cued recall test, trial-level accuracy (1 = correct, 0 = incorrect) served as the dependent variable. For the 30-day and 50-day delayed old/new recognition tests, accuracy was aggregated as the number of correct responses out of 15 items per condition per participant. Because each participant contributed only one aggregated value per condition, the models included random intercepts for participants but no random slopes to avoid over-parameterization.

Participants’ judgments of learning (JOLs)—the predicted number of correct responses out of 15 per condition and interval—were analyzed using the same GLMM specification. Fixed effects were learning condition (retrieval practice vs. repeated studying), retention interval (15 min, 30 days, 50 days), and their interaction. Only participants with complete JOL data across all three intervals were included (*n* = 39).

All models were fitted using the bobyqa optimizer with a maximum of 100,000 iterations; they converged successfully (convergence code = 0) and showed no singular fit issues (no zero variance estimates or perfect correlations among random effects).

## 3. Results

### 3.1. Fifteen-Minute Delayed Cued Recall Test Results

**Figure 2:**
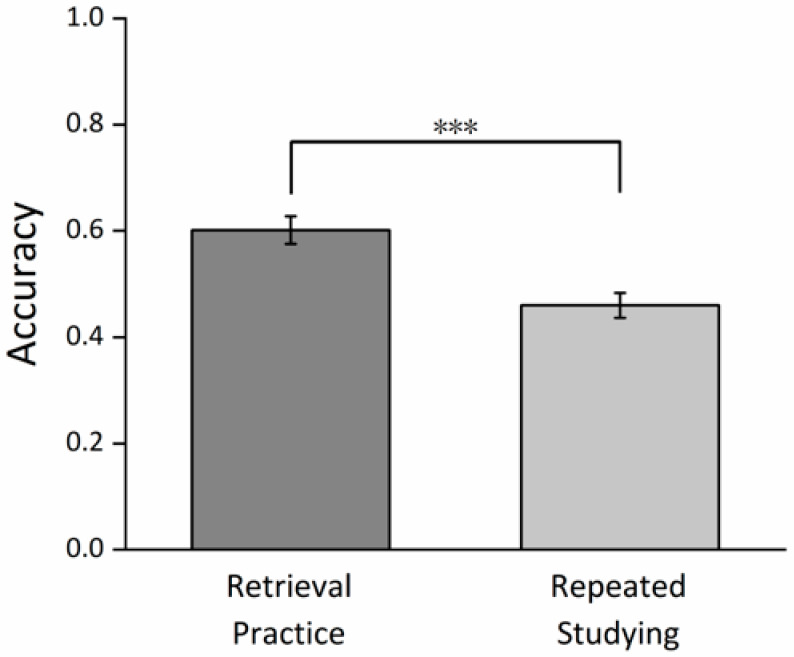
Results of the 15-min delayed cued recall test. The graph shows a significant difference between the retrieval practice condition and the repeated studying condition (*** *p* < 0.001). Error bars represent standard errors.

Figure 2 shows the proportion of Japanese words correctly recalled in the 15-min delayed cued recall test. The accuracy data were analyzed using a generalized linear mixed-effects model with the glmer function from the lme4 package (Bates et al., 2015). The proportion of correctly recalled words served as the dependent variable, with correct answers coded as 1 and incorrect answers coded as 0. Learning condition served as a fixed effect, with a random intercept for participants (items were not included as a random factor because each word appeared in only one condition). The main effect of the learning condition was statistically significant (χ^2^(1) = 33.28, *p* < 0.001). As shown in Figure 2, the retrieval practice condition resulted in a significantly higher proportion of correctly recalled words compared to the repeated studying condition (estimate = 0.60, SE = 0.10, z = 5.74, *p* < 0.001), corresponding to an odds ratio of 1.82 (95% CI: 1.47, 2.22), indicating that retrieval practice with feedback enhanced Japanese vocabulary learning.

### 3.2. Thirty-Day Delayed Old/New Recognition Memory Test Results

**Figure 3:**
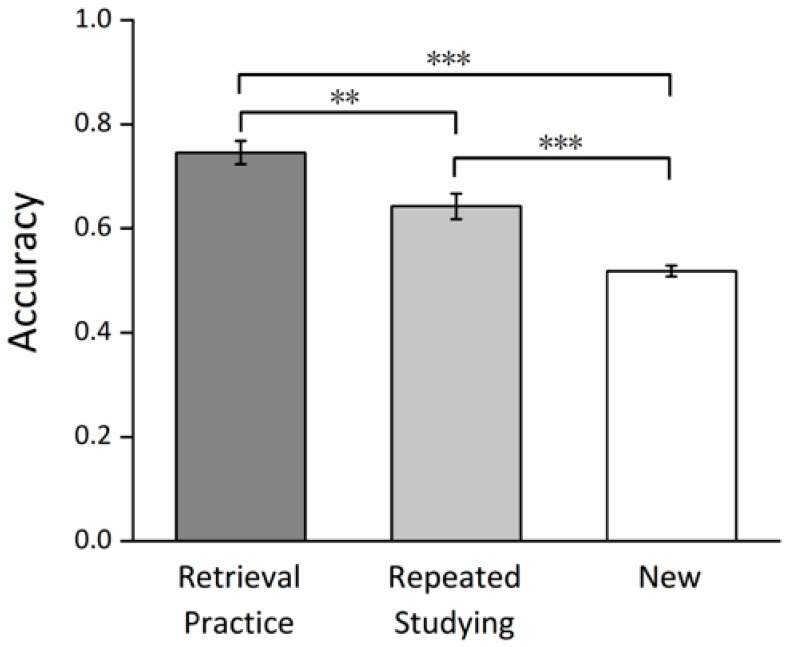
Results of the 30-day delayed old/new recognition memory test. The graph shows a highly significant difference between the retrieval practice condition and the repeated studying condition (** *p* < 0.01), and between the retrieval practice condition and the new word condition (*** *p* < 0.001). Additionally, repeated studying significantly outperformed the new word condition (*** *p* < 0.001). Error bars represent standard errors.

Figure 3 shows the recognition percentage of Japanese words in the 30-day delayed old/new recognition memory test. Recognition percentages for retrieval practice, repeated studying, and new words are presented separately. Table 2 provides specific information, with recognition percentage of 74.6% for retrieval practice condition, 64.2% for repeated studying condition, and 51.9% for new word condition. The recognition accuracy data were fitted to a generalized linear mixed-effects model. The likelihood ratio test (LRT) revealed a highly significant main effect of learning condition (χ^2^(2) = 64.79, *p* < 0.001). Post hoc comparisons indicated that retrieval practice was superior to new words (estimate = 1.04, SE = 0.14, z = 7.59, *p* < 0.001), corresponding to an odds ratio of 2.82 (95% CI: 2.16, 3.68). Retrieval practice was also superior to repeated studying (estimate = 0.51, SE = 0.16, z = 3.24, *p* = 0.0034), with an odds ratio of 1.66 (95% CI: 1.22, 2.25). Additionally, repeated studying was superior to new words (estimate = 0.53, SE = 0.13, z = 4.14, *p* < 0.001), with an odds ratio of 1.70 (95% CI: 1.32, 2.17).

### 3.3. Fifty-Day Delayed Old/New Recognition Memory Test Results

**Figure 4:**
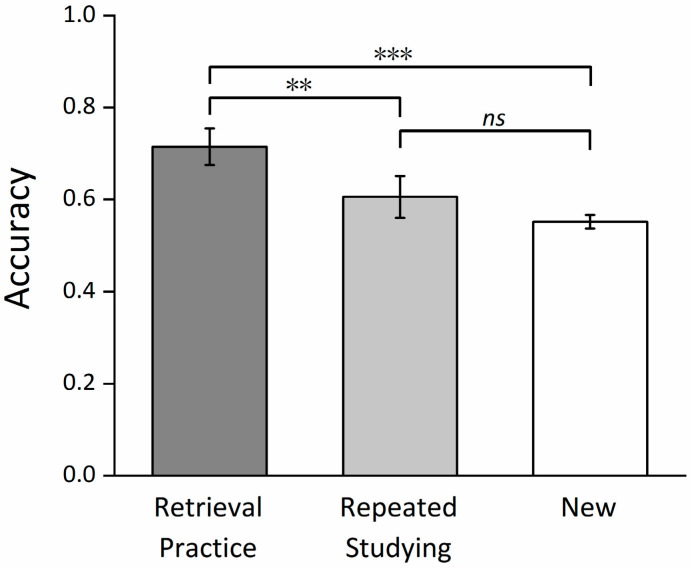
Results of the 50-day delayed old/new recognition memory test. The graph shows a highly significant difference between the retrieval practice condition and the repeated studying condition (** *p* < 0.01), and between the retrieval practice condition and the new word condition (*** *p* < 0.001). However, the repeated studying condition did not differ significantly from the new word condition (ns). Error bars represent standard errors.

The results of the 50-day delayed test are presented in Figure 4. Following the removal of outliers, data from 22 participants were included in the analysis. Similarly, the stimuli comprised three distinct conditions, and their corresponding recognition percentages are presented separately. Table 3 provides specific information, with a recognition percentage of 71.5% for the retrieval practice condition, 60.6% for the repeated studying condition, and 55.2% for the new word condition. The recognition accuracy data were fitted to a generalized linear mixed-effects model. The likelihood ratio test (LRT) revealed a highly significant main effect of learning condition (χ^2^(2) = 25.91, *p* < 0.001). Post hoc comparisons indicated that retrieval practice was superior to new words (estimate = 0.73, SE = 0.15, z = 4.99, *p* < 0.001), corresponding to an odds ratio of 2.08 (95% CI: 1.54, 2.78). Retrieval practice was also superior to repeated studying (estimate = 0.50, SE = 0.17, z = 2.99, *p* = 0.0079), with an odds ratio of 1.65 (95% CI: 1.19, 2.29). However, new words did not differ significantly from repeated studying (estimate = −0.23, SE = 0.14, z = −1.66, *p* = 0.2227), with an odds ratio of 0.80 (95% CI: 0.60, 1.06).

### 3.4. Metacognitive Learning Judgment Results

**Figure 5:**
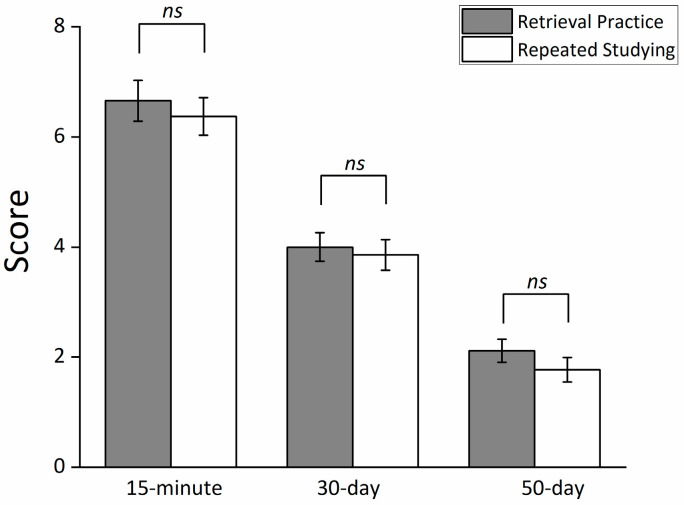
Results of metacognitive judgments of learning (JOLs) for the 15-min, 30-day, and 50-day delays. Black bars represent the retrieval practice condition; white bars represent the repeated studying condition. The graph shows no significant difference between the two learning conditions at any retention interval (ns). Error bars represent standard errors.

Figure 5 displays the participants’ judgments of learning (JOLs)—the average predicted number of correct responses for each retention interval (15 min, 30 days, 50 days) and learning condition (retrieval practice, RP; repeated studying, RS). A generalized linear mixed-effects model (binomial family, logit link) was fitted with learning condition, retention interval, and their interaction as fixed effects, and a random intercept for participants. The analysis revealed a significant main effect of retention interval (χ^2^(2) = 114.2, *p* < 0.001, derived from the fixed effects of time 30 days and time 50 days), indicating that the predicted performance decreased over longer delays. The main effect of learning condition was not significant (χ^2^(1) = 0.51, *p* = 0.475). The interaction between condition and interval was also not significant (χ^2^(2) = 0.35, *p* = 0.838). Post hoc comparisons showed that at each retention interval, there was no significant difference between RP and RS (15 min: OR = 0.96, z = −0.37, *p* = 0.715; 30 days: OR = 0.98, z = −0.14, *p* = 0.893; 50 days: OR = 0.87, z = −0.85, *p* = 0.397). Thus, while the participants correctly anticipated that memory would decline over time, they did not predict any advantage of retrieval practice over repeated studying, even at the longest delay.

Additionally, the high attrition rate (46.6% at 30 days, 62.1% at 50 days) may have introduced self-selection bias. Following J. R. Carpenter and Smuk (2021), worst-case sensitivity analyses revealed that under extreme missing-data assumptions (dropouts performing at chance level or at the completers’ new-word mean), the advantage of retrieval practice over repeated studying became non-significant for both the 30-day and 50-day tests (*p* = 0.068 and *p* = 0.075, respectively), indicating that this comparison is sensitive to attrition.

## 4. Discussion

This research employed a mixed design to investigate the impact of learning methods on native Chinese speakers acquiring Japanese vocabulary using the retrieval practice paradigm, comparing short-term and long-term memory performance between retrieval practice and repeated studying. Results revealed that for Japanese vocabulary written in kana, participants in the retrieval practice condition demonstrated higher levels of vocabulary recall in the 15-min delayed cued recall test and higher recognition accuracy in both the 30-day and 50-day delayed old/new recognition tests relative to repeated studying. This outcome demonstrates the advantage of retrieval practice in Chinese native speakers’ Japanese language acquisition, consistent with previous research on the retrieval practice effect, including L2 and L3 contexts.

Specifically, in the 15-min delayed recall test, participants performed significantly better in the retrieval practice condition compared to the repeated studying condition. This finding extends previous research on European languages to Japanese vocabulary learning, suggesting the existence of the retrieval practice effect in Japanese acquisition, as retrieval practice consistently produces better memory performance (Candry et al., 2020; Karpicke & Roediger, 2008; Li et al., 2022a). Notably, in the present study, participants in the retrieval practice condition engaged in productive learning (L1 to L3), rather than receptive learning methods (L3 to L1), implying higher cognitive effort, as participants needed to remember the specific strokes of Japanese vocabulary rather than just the general shape. This design aimed to widen the gap in cognitive effort between learning conditions. As expected, the results showed a significant retrieval practice effect, with conditions requiring greater cognitive and attentional resources leading to superior memory performance. These observed advantages align with the predictions of the retrieval effort hypothesis (Pyc & Rawson, 2009), which posits that cognitively effortful retrieval strengthens memory traces more effectively than passive restudying. Similarly, the finding that more demanding tasks facilitated learning is also consistent with the desirable difficulty framework (Bjork, 1994; Pyc & Rawson, 2009), which proposes that moderate difficulty during learning promote deeper understanding and long-term memory formation. These results suggest that the retrieval practice effect can be observed in both alphabetic and logographic scripts within the present experimental context. However, direct comparisons and neuroimaging evidence are needed to draw stronger conclusions about the underlying lexical processing pathways.

The choice of kana materials also provides theoretical insights. Learning kana, a completely unfamiliar syllabary, requires a high level of cognitive effort, which directly tests the retrieval effort hypothesis (Pyc & Rawson, 2009). The finding that retrieval practice outperformed repeated study under such demanding conditions supports the claim that effortful retrieval strengthens memory traces, even when the material lacks any semantic or orthographic familiarity. Although kana itself has no inherent meaning, some participants reported in the post-experiment debriefing that they spontaneously generated visual or semantic associations (e.g., one participant linked the shape of “へ” to a roof) during retrieval attempts. This provides indirect evidence for the elaborative retrieval account (S. K. Carpenter, 2009), suggesting that retrieval practice encourages the formation of rich, idiosyncratic cues that facilitate later access, even for visually complex stimuli. Moreover, by examining L3 acquisition in native Chinese speakers (with English as L2), the present study shows that retrieval practice effects remain robust in a multilingual context where the target language (Japanese kana) shares no orthographic similarity with either L1 or L2. This extends prior research beyond L2 settings and supports the cross-linguistic generality of the testing effect, while acknowledging the need for further replication with other language pairings.

Moreover, while the desirable difficulty framework primarily emerged from studies on alphabetic languages, this study extends it to a phonographic L3 script (Japanese kana) in native Chinese speakers (with English as L2). Consistent with prior work on L3 learning (e.g., Li et al., 2022b), our findings offer preliminary evidence that retrieval practice effects may be robust across typologically different language pairings, even when the target script lacks any orthographic resemblance to the learner’s existing languages. In that study (Li et al., 2022b), L3 (French, alphabetic) was typologically similar to L2 (English) but dissimilar to L1 (Chinese, logographic). The present study examines a complementary, more controlled scenario: Chinese native speakers learning Japanese as an L3. Although Japanese and Chinese share logographic kanji characters, we deliberately employed kana materials—a phonographic script entirely unfamiliar to these learners and orthographically distinct from both Chinese characters and the English alphabet. By isolating kana, we dissociated the retrieval practice effect from L1-mediated positive transfer and orthographic parasitism, thereby testing whether retrieval practice remains advantageous when the target script diverges substantially from both L1 and L2. The observed behavioral advantages suggest that retrieval practice effects can be observed across typologically different language pairings even under these more stringent conditions, although this conclusion remains tentative given the limited range of scripts and populations examined. Future research should conduct direct cross-linguistic comparisons to test the generalizability of these findings.

Furthermore, we emphasize the use of kana-written words, which reduce positive transfer from Chinese to Japanese. As noted earlier, Japanese kanji vocabulary shares high typological similarity with Chinese, and may lead to cognitive phenomena known as parasitic lexical associations that disrupt participants’ learning performance, while kana vocabulary acquisition can eliminate this effect to a large extent. This design not only mitigated transfer interference but also posed greater challenges requiring increased attention and effort, and yielded improved learners’ vocabulary proficiency (Kato, 2005; Mariko et al., 2001). Additionally, with increasing kana usage in contemporary Japanese literature and culture, learning Japanese through kana aligns with current trends. However, given the artificial laboratory task with 30 isolated word pairs, the findings may not directly translate to authentic classroom instruction. Any pedagogical implications should be considered preliminary and tested in ecologically valid educational settings.

As a speculative pedagogical implication, even covert retrieval (silent mental recall) can be effectively implemented in classroom settings. For example, teachers can pause during a lesson and ask students to silently recall the Japanese kana word for a given Chinese prompt, then provide immediate corrective feedback by displaying the correct answer. This activity only takes a few seconds, avoids logistical issues of handwriting or typing, and can be integrated into regular quizzes or digital flashcards. Learners can also use covert retrieval for self-testing: after seeing a cue, they try to retrieve the target form in their minds before checking the answer. These simple techniques transform passive review into active retrieval, potentially enhancing long-term retention without disrupting class flow.

Importantly, our research examined retention effects after 30 days and 50 days, revealing that participants in the retrieval practice condition achieved higher recognition rates on the old/new recognition memory test. Similar results have been identified in previous studies (Roediger & Karpicke, 2006; Karpicke & Roediger, 2008; S. K. Carpenter et al., 2009). Karpicke and Roediger (2008) demonstrated that learning can assist in initial encoding, but contributes little to further deepening memory traces. In contrast, retrieval requires the reintegration and reorganization of memory content, hence promoting memory consolidation and long-term retention. Critically, our paradigm incorporated retrieval practice coupled with corrective feedback—a mechanism empirically demonstrated to augment learning motivation and counteract memory decay through error-driven reconsolidation (Li et al., 2022b). Specifically, corrective feedback helps learners revise incorrect memory traces, reinforce correct representations, and form more solid memory traces with this updated information. This aligns with the retrieval effort hypothesis (Pyc & Rawson, 2009), which posits that the cognitive effort expended during successful retrieval strengthens memory traces, thus potentiating long-term retention. Moreover, corrective feedback guides learners to correct their mistakes, promoting greater cognitive effort and resulting in effective memory retention. Importantly, as we mentioned above, few investigations have examined retention periods exceeding one month, and our findings reveal that retrieval practice maintains a statistically significant memory advantage even at extended intervals of 50 days. However, it should also be noted that the delayed test may also induce practice effects, so that the long-term retention observed after 50 days could potentially be attributed to repeated retrieval during prior testing sessions.

This study provides behavioral evidence for the advantages of retrieval practice in Chinese native speakers’ Japanese acquisition through the classic retrieval practice paradigm, demonstrating that retrieval practice with feedback significantly enhances both short-term and long-term memory retention compared to repeated studying. Consistent with prior work, participants did not perceive a reliable advantage of retrieval practice over repeated studying at the 15-min or 30-day intervals. Rivers (2021) noted that learners often prefer repeated studying, possibly because they cannot accurately monitor their progress during retrieval practice, leading to skepticism about its effectiveness. Similarly, teachers frequently fail to recognize the significance of retrieval practice, often viewing testing merely as a tool for monitoring outcomes rather than as a learning device (Piza et al., 2019). In the present data, no significant difference in JOLs was observed between the two learning conditions at any retention interval, including the 50-day delay. This null effect is consistent with the overall pattern reported in Section 3.4. The absence of a JOL advantage aligns with the metacognitive illusion that effortful retrieval signals poorer learning, even when objective performance shows the opposite (Bjork, 1994). Given the reduced sample size at 50 days (*n* = 22) and the absence of any interaction or simple effect in the statistical analysis, there is no evidence that metacognitive sensitivity improves over longer delays under the present paradigm. Future studies should collect JOLs at multiple time points, use calibration measures, and preregister analyses to better understand how retrieval practice affects metacognitive monitoring. Nonetheless, as Rivers (2021) observed, students may initially intend to use effective strategies but procrastinate and ultimately fall back on less effective approaches. A persistent gap remains between the empirical support for retrieval practice and its widespread adoption in actual learning contexts.

Moreover, worst-case sensitivity analyses indicated that the advantage of retrieval practice over repeated studying was not robust to extreme missing-data assumptions. Under the most conservative scenarios (assuming dropouts performed at chance level or at the completers’ new-word mean), the RP versus RS comparison lost statistical significance at both the 30-day and 50-day tests. Therefore, while retrieval practice clearly outperformed new words, its superiority over repeated studying may be inflated by participant attrition. These caveats should be acknowledged when interpreting the long-term mnemonic benefits of retrieval practice relative to repeated studying. While the observed differences are statistically significant, the practical magnitude of the retrieval practice advantage remains to be established, especially with respect to the meaningful vocabulary knowledge that learners can actively produce. The odds ratios reported indicate moderate effects, but educational significance should be assessed in real-world contexts.

We also acknowledge several limitations. The sample was modest and homogeneous (all native Chinese speakers with English as L2), which limits generalizability to other L1–L3 pairings. Participant attrition was high (53.4% at 30 days, 62.1% at 50 days), potentially introducing self-selection bias; sensitivity analyses indicated that the advantage of retrieval practice over repeated studying was not robust to extreme missing-data assumptions, suggesting that this long-term benefit may be inflated by dropout. Notably, the laboratory setting with isolated word pairs differs considerably from authentic classroom instruction. Moreover, although recognition tests are more sensitive to residual memory after long delays, they do not directly measure productive vocabulary knowledge (e.g., cued recall or free production), so the observed advantage may not fully capture the kind of active word use required in real-world language learning. The study also only used nouns and kana vocabulary, leaving verbs, adjectives, and kanji untested. Additionally, retrieval practice was purely covert (no writing or typing), whereas the final test required handwritten recall without prior handwriting practice, which may have added variability. Finally, the fixed 5-s retrieval window could induce time-pressure errors unrelated to learning, and treating uncertain responses (space bar) as incorrect—though conservative—may underestimate true recognition. Future studies should address these limitations by using larger and more diverse samples, reducing attrition, testing logographic scripts and other word classes, conducting classroom-based replications, including both recognition and recall measures, and adopting longer or self-paced retrieval windows.

## 5. Conclusions

The present study demonstrates that retrieval practice with corrective feedback significantly enhances short-term (15-min) cued recall and long-term (30-day and 50-day) recognition of L3 Japanese kana vocabulary in native Chinese speakers. These results support the retrieval effort hypothesis and the desirable difficulty framework in a novel phonographic learning context.

The findings, however, are constrained by several limitations. The sample size was modest and homogeneous, the attrition rate was high, and the study used only nouns and kana vocabulary. In addition, the laboratory setting with isolated word pairs differs from authentic classroom instruction, raising concerns about ecological validity. The fixed 5-s retrieval window may also have been insufficient for some participants.

Future research should explore the generalizability of these effects to logographic (kanji) learning, examine other L1–L3 pairings, and conduct classroom-based replication studies. Neuroimaging methods could also be employed to uncover the neural mechanisms underlying retrieval practice in unfamiliar script acquisition.

The present findings were derived from a laboratory task using isolated word pairs; thus, direct generalization to authentic classroom contexts should be made cautiously. Nevertheless, the current results support the benefit of retrieval practice for third language learning, and learners may use self-testing to enhance memory retention. Furthermore, even covert retrieval can be easily implemented into classroom activities and self-study routines. Notably, retrieval practice remains underutilized in real-world classrooms, and advancing its educational uptake remains an important challenge.

## Figures and Tables

**Table 1:** Descriptive statistics of frequency and stroke numbers for Chinese and Japanese in two learning conditions (retrieval practice and repeated studying).

	Chinese		Japanese	
Learning Condition	Frequency	Stroke Numbers	Frequency	Stroke Numbers
Retrieval Practice	3.09 (0.45)	15.87 (4.72)	3.53 (0.39)	5.67 (1.99)
Repeated Studying	3.05 (0.40)	15.87 (5.49)	3.53 (0.39)	5.47 (1.55)

**Table 2:** Average recognition percentages (M) with standard deviations (SDs) and 95% confidence intervals (CIs) for retrieval practice, repeated studying, and new words on the 30-day delayed old/new recognition memory test (*n* = 27).

Condition	M (%)	SD	95% CI (%)
Retrieval Practice	74.57	0.15	[68.63, 80.51]
Repeated Studying	64.20	0.19	[56.68, 71.72]
New Words	51.85	0.10	[47.89, 55.81]

**Table 3:** Average recognition percentages (M) with standard deviations (SDs) and 95% confidence intervals (CIs) for retrieval practice, repeated studying, and new words on the 50-day delayed old/new recognition memory test (*n* = 22).

Condition	M (%)	SD	95% CI (%)
Retrieval Practice	71.52	0.19	[63.09, 79.95]
Repeated Studying	60.61	0.21	[51.62, 69.60]
New Words	55.15	0.07	[52.05, 58.25]

## Data Availability

The datasets generated during and/or analyzed during the current study are available from the corresponding author on request.
